# Comparison between gastrostomy feeding and self-expandable metal stent insertion for patients with esophageal cancer and dysphagia

**DOI:** 10.1371/journal.pone.0179522

**Published:** 2017-06-20

**Authors:** Yang Won Min, Eun Young Jang, Ji Hey Jung, Hyuk Lee, Byung-Hoon Min, Jun Haeng Lee, Poong-Lyul Rhee, Jae J. Kim

**Affiliations:** Department of Medicine, Samsung Medical Center, Sungkyunkwan University School of Medicine, Seoul, Korea; University Hospital Llandough, UNITED KINGDOM

## Abstract

**Background:**

Self-expandable metal stent (SEMS) insertion and percutaneous gastrostomy (PG) feeding are commonly used for patients with esophageal cancer and dysphagia. This study aimed to compare outcomes between SEMS insertion and PG feeding for them.

**Methods:**

We retrospectively analyzed 308 patients with esophageal cancer who underwent fully covered SEMS insertion (stent group) or PG (gastrostomy group) for dysphagia due to tumor. Patients with other causes of dysphagia, such as radiation-induced or postoperative stricture, were excluded from the study. Clinical outcomes were compared between the two groups, including overall survival and need for additional intervention and postprocedural nutritional status.

**Results:**

At baseline, the stent group (n = 169) had more stage IV patients, less cervical cancers, and received radiotherapy and esophagectomy less often than the gastrostomy group (n = 64). The Kaplan-Meier curves showed higher overall survival in the gastrostomy group than in the stent group. Multivariate analysis revealed that PG was associated with better survival compared with SEMS insertion (hazard ratio 0.541, 95% confidence interval 0.346–0.848, p = 0.007). In addition, the gastrostomy group needed additional intervention less often (3.1% vs. 21.9%, p < 0.001) and experienced less decrease in serum albumin levels (-0.15 ± 0.56 g/dL vs. -0.39 ± 0.58 g/dL, p = 0.011) than the stent group after procedure.

**Conclusions:**

Our data suggested that, compared with SEMS insertion, PG is associated with better overall survival in patients with esophageal cancer and dysphagia. Stabilized nutritional status by PG may play a role in improving patient survival.

## Introduction

The incidence of esophageal cancer is increasing, and esophageal cancer ranked ninth for cancer incidence and sixth for cancer death in 2013 [1]. Many patients with esophageal cancer are diagnosed at an advanced stage, and dysphagia is their predominant symptom Fully or partially covered self-expandable metal stent (SEMS) placement for palliation of dysphagia caused by esophageal cancer has become the standard of care because of its better efficacy compared to other treatment modalities [2,3]. Indeed, rapid palliation of dysphagia is a major advantage of SEMS insertion [4,5]. However, SEMS has a risk of adverse events such as recurrent dysphagia, pain, gastroesophageal reflux, and esophagorespiratory fistula [6]. Thus, the quality of life (QoL) decreases immediately following treatment in patients who receive SEMS insertion [7].

Patients with esophageal cancer are more likely to experience weight loss and have a higher risk of malnutrition than patients with other cancers [8]. Although dysphagia caused by obstruction is the main cause of malnutrition, patients with esophageal cancer frequently have compromised oral intake during treatment with or without curative intent. As weight loss and malnutrition are determinants of tolerance to treatment and survival [9], not only restoring swallowing but also maintaining nutritional status should be pursued in patients with esophageal cancer and dysphagia. Percutaneous gastrostomy (PG) feeding can provide optimal nutritional support and stabilize QoL in patients with cancer and dysphagia [10,11]. Thus, currently SEMS and PG have been commonly used to maintain nutrition in patients with malignant dysphagia due to esophageal cancer. However, no direct comparison between SEMS insertion and PG feeding has been performed, even in a retrospective design. The present study aimed to compare the efficacy of fully covered SEMS (FCSEMS) insertion and PG feeding in terms of clinical outcomes, including overall survival and nutritional status, in patients with esophageal cancer and dysphagia.

## Methods

### Study population

**Fig 1:**
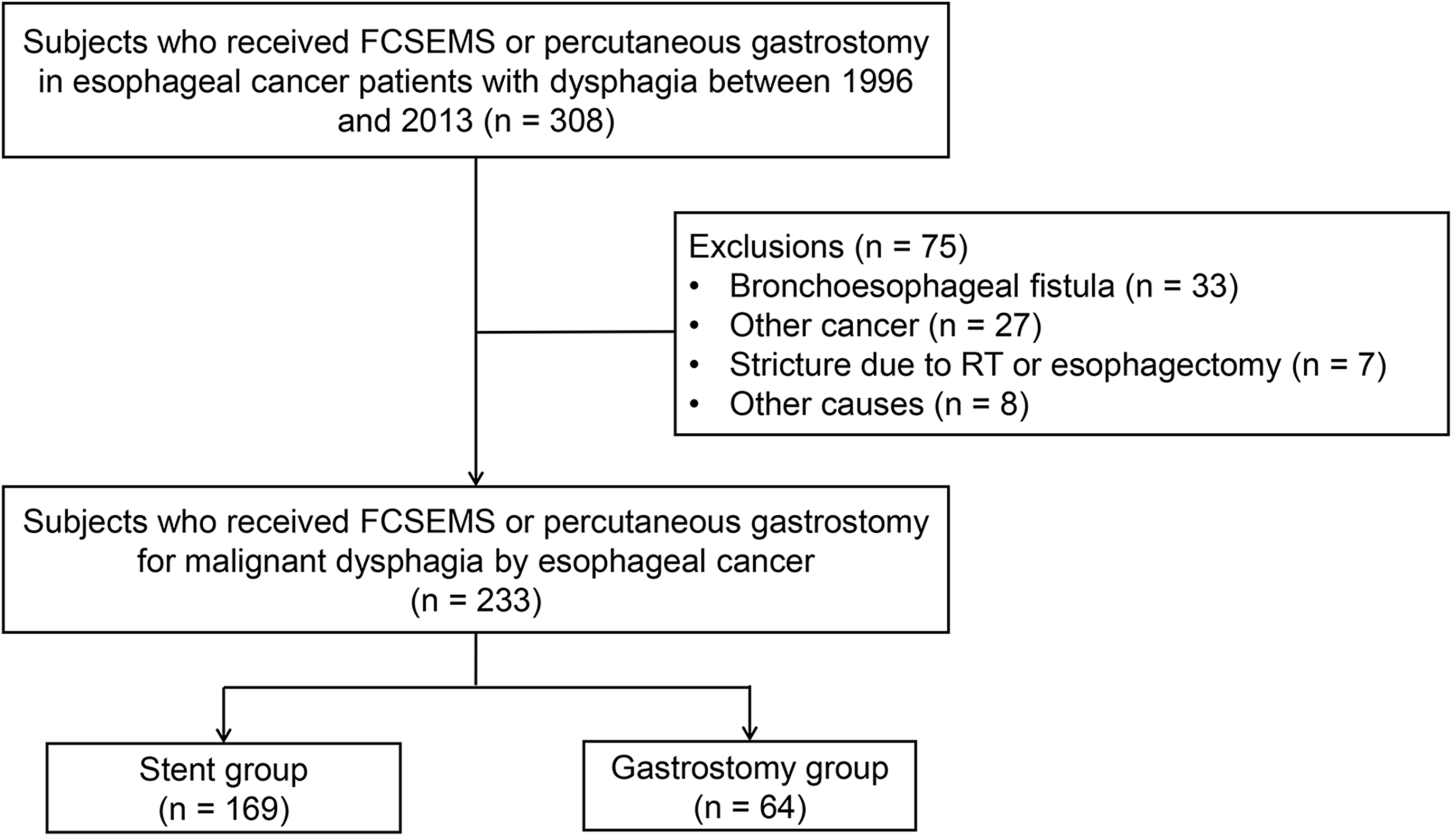
Flow chart of patient selection. FCSEMS, fully covered self-expandable metal stent; RT, radiotherapy.

We retrospectively analyzed a total of 308 patients with esophageal cancer who underwent FCSEMS insertion or PG for dysphagia at the Samsung Medical Center between January 1996 and December 2013. The diagnosis of esophageal cancer was made by histological confirmation. Procedures were endoscopically or radiologically performed. Although there were not specific indications for FCSEMS insertion or PG, FCSEMS insertion was preferred to patients who wanted to continue oral intake and PG was often performed for cervical cancers. Patients were excluded from the study if they met the following criteria: 1) bronchoesophageal fistula (n = 33), 2) other cancer (n = 27), 3) stricture due to operation or radiotherapy (n = 7), 4) underwent the procedure previously at another hospital (n = 3), 5) recurrent cancer after radiotherapy and esophagectomy (n = 2), 6) lye stricture (n = 1), 7) underwent jejunostomy prior to the procedure (n = 1), or 8) were lost to follow-up (n = 1). Finally, 169 patients who underwent FCSEMS insertion (stent group) and 64 patients who received PG feeding (gastrostomy group) were included in the analysis (Fig 1). This study protocol was reviewed and approved by the Institutional Review Board of the Samsung Medical Center (No. 2015-12-030). The board waived the requirement for informed consent.

### Data collection and definitions

The following data was collected as baseline characteristics: age, sex, tumor stage according to AJCC 7^th^ [12], tumor histology, tumor location, length of obstruction by tumor (determined by endoscopy, esophagography, or computed tomography [CT]), history of chemotherapy, radiotherapy, or esophagectomy before the procedure, body weight, and serum albumin level (S1 File). Data regarding the occurrence of procedure-related complications, presence of aspiration pneumonia, treatment with chemotherapy, radiotherapy, or esophagectomy, and changes of body weight and serum albumin level after the procedure were also obtained. Changes in weight and albumin level were determined by the values measured 1 to 2 months after the procedure. Aspiration pneumonia was defined when a patient had a history of aspiration according to medical records and had a chest CT scan showing gravity-dependent opacity, which is known to be indicative of aspiration pneumonia. Survival and the presence of additional intervention following the procedure (FCSEMS insertion or PG) were also investigated. Additional intervention included stent insertion, stent reposition, gastrostomy, and removal of stent or gastrostomy due to complications.

### Outcomes

All-cause mortality was investigated for survival, which was the primary outcome. Secondary outcomes were the need for additional intervention, number of additional interventions, presence of complications, presence of aspiration pneumonia, and change in body weight or serum albumin level after the procedure.

### Statistical analysis

Data are shown as the mean ± SD or number (%) of patients. We used the Kaplan-Meier method to obtain survival curves and the log-rank test to assess the differences in the survival curves. Baseline characteristics and secondary outcomes were compared between the two groups by using the t-test, chi-square test, and Fisher’s exact test. Cox proportional hazards models were used to calculate the hazard ratios (HRs) for each type of procedure (FCSEMS or PG) after adjustment for the other baseline characteristics. A *P* value of less than 0.05 was considered to be significant. SAS ver. 9.4 software (SAS Institute, Cary, NC, USA) was used for all analyses.

## Results

### Baseline characteristics

Table 1 shows the comparison of baseline characteristics between the two groups. The mean age of patients in the stent and gastrostomy groups was 64.5 ± 10.2 and 62.3 ± 10.2 years, respectively (p = 0.141). The stent group had more men (93.5% vs. 78.1%, p < 0.001) and stage IV patients (88.8% vs. 57.8%, p < 0.001) than the gastrostomy group. Tumor location was different between the two groups (p < 0.001); lower thoracic obstruction was the most common in the stent group (42.6%) while cervical obstruction was most common in the gastrostomy group (35.9%). However, tumor histology and length of obstruction by the tumor did not differ between the two groups. The gastrostomy group received radiotherapy before and after the procedure (42.2% vs. 20.7% and 37.5% vs. 20.7%, respectively) and esophagectomy after procedure (20.3% vs. 4.7%) more often than the stent group (p < 0.001).

**Table 1:** Comparison of baseline characteristics between esophageal cancer patients who received either esophageal self-expanding metal stent or percutaneous gastrostomy for malignant dysphagia.

Variables	Stent group (n = 169)	Gastrostomy group (n = 64)	p
Age (years)	64.5 ± 10.2	62.3 ± 10.2	0.141
Sex (male)	158 (93.5)	50 (78.1)	< 0.001
Stage			< 0.001
II+III	19 (11.2)	27 (42.2)	
IV	150 (88.8)	37 (57.8)	
Location			< 0.001
Cervical	5 (3.0)	23 (35.9)	
Upper thoracic	33 (19.5)	17 (26.6)	
Mid thoracic	59 (34.9)	7 (10.9)	
Lower thoracic	72 (42.6)	17 (26.6)	
Histology			0.290
Squamous cell cancer	156 (94.6)	63 (98.4)	
Adenocarcinoma	13 (5.4)	1 (1.6)	
Obstruction length (cm)	6.50 ± 2.76	5.94 ± 2.94	0.178
Chemotherapy			0.063
None	39 (23.1)	11 (17.2)	
Before procedure	90 (53.3)	28 (43.8)	
After procedure	40 (23.7)	25 (39.1)	
Radiotherapy			< 0.001
None	99 (58.6)	13 (20.3)	
Before procedure	35 (20.7)	27 (42.2)	
After procedure	35 (20.7)	24 (37.5)	
Esophagectomy			< 0.001
None	161 (95.3)	51 (79.7)	
Before procedure	0 (0)	0 (0)	
After procedure	8 (4.7)	13 (20.3)	

Data are shown as the mean ± SD or number (%) of patients.

### Overall survival

**Fig 2:**
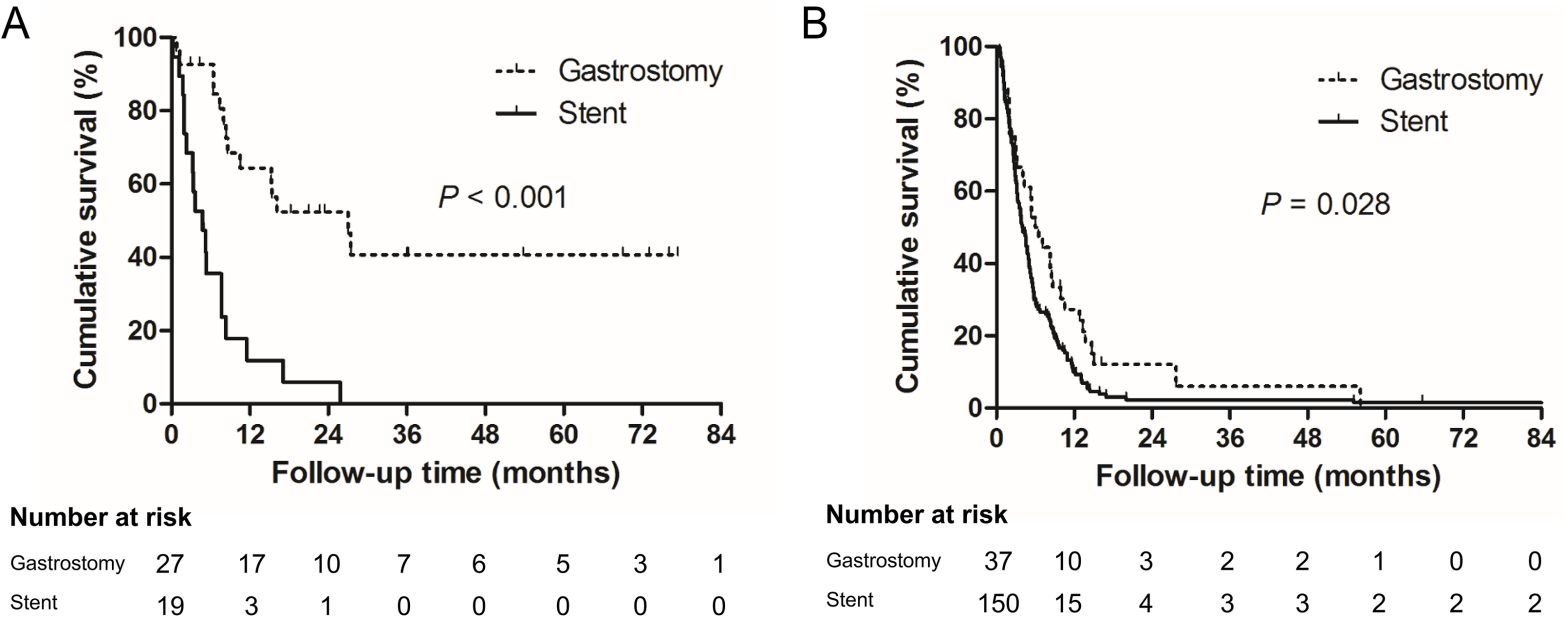
(A) Kaplan-Meier curves for overall survival in stage II and III esophageal cancer patients who received either esophageal self-expanding metal stent or percutaneous gastrostomy for malignant dysphagia. (B) Kaplan-Meier curves for overall survival in stage IV esophageal cancer patients who received either esophageal self-expanding metal stent or percutaneous gastrostomy for malignant dysphagia.

During the median follow up of 4.9 months (interquartile range 2.4–9.6 months; maximum 145.7 months), the Kaplan-Meier curves revealed significantly superior survival in the gastrostomy group compared to the stent group (stage II+III, p < 0.001 and stage IV, p = 0.028, Fig 2). Multivariate analysis showed that treatment with chemotherapy before or after the procedure, treatment with radiotherapy before the procedure, treatment with esophagectomy after the procedure, and the type of procedure (FCSEMS insertion vs. PG) were independent prognostic factors associated with overall survival (Table 2). The gastrostomy group showed better survival than the stent group with an HR of 0.557 (95% confidence interval 0.358–0.867, p = 0.007).

**Table 2:** Prognostic factors associated with overall survival in patients with esophageal cancer and dysphagia.

Variables	Univariate analysis		Multivariate analysis	
	HR (95% CI)	p	HR (95% CI)	p
Age (years)	1.012 (0.999–1.025)	0.079	0.984 (0.968–1.001)	0.061
Sex				
Male	1		1	
Female	0.431 (0.261–0.714)	0.001	0.581 (0.334–1.009)	0.054
Stage				
II+III	1		1	
IV	2.552 (1.733–3.760)	< 0.001	1.201 (0.752–1.916)	0.443
Location				
Cervical	1		1	
Upper thoracic	2.045 (1.201–3.482)	0.008	1.406 (0.788–2.507)	0.249
Mid thoracic	3.292 (1.968–5.506)	< 0.001	1.642 (0.890–3.028)	0.113
Lower thoracic	2.218 (1.357–3.626)	0.002	1.361 (0.757–2.446)	0.303
Obstruction length (cm)	1.039 (0.994–1.086)	0.090	1.035 (0.983–1.089)	0.188
Chemotherapy				
None	1		1	
Before procedure	0.776 (0.550–1.095)	0.149	0.548 (0.352–0.851)	0.008
After procedure	0.434 (0.293–0.644)	< 0.001	0.453 (0.280–0.733)	0.001
Radiotherapy				
None	1		1	
Before procedure	0.953 (0.681–1.334)	0.779	1.708 (1.158–2.519)	0.007
After procedure	0.638 (0.455–0.894)	0.009	0.974 (0.674–1.406)	0.888
Esophagectomy				
None	1		1	
After procedure	0.249 (0.141–0.442)	< 0.001	0.281 (0.148–0.533)	< 0.001
Procedure				
Stent	1		1	
Gastrostomy	0.393 (0.281–0.550)	< 0.001	0.557 (0.358–0.867)	0.010

HR, hazard ratio; CI, confidence interval

### Secondary outcomes

Table 3 shows the comparison of secondary outcomes between the two groups. The stent group needed additional intervention more often than the gastrostomy group (21.9% vs. 3.1%, p < 0.001). In addition, the number of additional interventions was higher in the stent group than in the gastrostomy group (0.27 ± 0.55 times vs. 0.05 ± 0.28 times, p < 0.001). There was no significant difference in the presence of procedure-related complication or aspiration pneumonia. Although change in body weight after the procedure did not differ between the two groups, albumin level after the procedure dropped more in the stent group than in the gastrostomy group (-0.39 ± 0.58 g/dL vs. -0.15 ± 0.56 g/dL, p = 0.011).

**Table 3:** Comparison of secondary outcomes between esophageal cancer patients who received either esophageal self-expanding metal stent or percutaneous gastrostomy for malignant dysphagia.

Variables	Stent group (n = 169)	Gastrostomy group (n = 64)	p
Need for additional intervention	37 (21.9)	2 (3.1)	< 0.001
Number of additional interventions (times)	0.27 ± 0.55	0.05 ± 0.28	< 0.001
Procedure-related complications	5 (3.0)	1 (1.6)	1.000
Aspiration pneumonia	26 (15.4)	11 (17.2)	0.737
Change in weight after the procedure (kg)	-0.61 ± 3.59	-0.36 ± 2.60	0.604
Change in serum albumin level after the procedure (g/dL)	-0.39 ± 0.58	-0.15 ± 0.56	0.011

Data are shown as the mean ± SD or number (%) of patients.

## Discussion

Currently, fully or partially covered SEMS insertion and PG have become the most common procedures to treat dysphagia in patients with esophageal cancer. However, the optimal procedure has not been established. In patients with advanced esophageal cancer, stabilized nutritional status is important for tolerance to treatment and survival. Thus, in terms of survival, the strength of PG feeding in providing optimal nutritional support is more important than the benefit of SEMS insertion in providing rapid palliation of dysphagia. The present study is the first to compare the two procedures. We observed that PG feeding was associated with better overall survival in patients with esophageal cancer and dysphagia compared with FCSEMS insertion. Furthermore, the gastrostomy group needed less additional interventions and experienced less decrease in serum albumin levels than the stent group. These observations indicate that stabilized nutritional status by PG feeding may play a role in improving survival in patients with esophageal cancer and dysphagia as compared to FCSEMS insertion.

Fully or partially covered SEMS insertion is recommended for palliation of malignant dysphagia [2]. Indeed, SEMS has shown superior efficacy for the palliation of malignant dysphagia in several randomized controlled trials (RCTs) compared to photodynamic therapy, laser therapy, esophageal bypass, and rigid plastic stents, and SEMS has improved the dysphagia score by at least 2 points within 1–2 days [3,13]. However, analysis of pooled data from RCTs and observational studies showed frequent adverse events associated with SEMS for malignant dysphagia [2]. Among them, severe pain was the most common and was reported in up to 35% of patients. The rate of pain after SEMS varies depending on the type of stent; the expansion force and decreased flexibility of the stent may play a role in the generation of pain [14–17]. In addition, gastroesophageal reflux occurs frequently, especially when the stent is placed across the esophagogastric junction, although there have been some reports showing the preventive effect of an antireflux stent [18,19]. These common adverse events may make it difficult to maintain oral food intake. Recurrent dysphagia because of stent migration, tumor and/or tissue ingrowth or overgrowth, and food impaction also could disrupt optimal nutritional support.

Malnutrition is a determinant of tolerance to treatment and survival in patients with esophageal cancer and is associated with a poor prognosis [20,21]. However, weight loss is reported in 79% of patients with esophageal cancer because of the increased metabolic demand and insufficient nutritional intake [22,23]. In addition to dysphagia and anorexia, esophagitis and side effects from radiotherapy and chemotherapy compromise oral intake. Therefore, patients with esophageal cancer could have persistent deterioration in nutritional status even after dysphagia is improved by SEMS insertion [24]. PG feeding can be used in patients with dysphagia and head and neck cancer and esophageal cancer to stabilize or improve the patients’ nutritional status [25,26]. PG is readily placed with limited complications using percutaneous endoscopic gastrostomy or percutaneous radiologic gastrostomy techniques, even in patients with advanced esophageal cancer [11,27,28]. Indeed, our results showed that PG feeding was associated with better survival and nutritional support as compared to SEMS insertion. Although changes in body weight after the procedure did not reach statistical significance between the two groups, serum albumin levels after the procedure dropped less in the gastrostomy group than in the stent group. However, nutritional status was determined 1 to 2 months after the procedure, which might be too short a time for decreased body stores to be evidenced by weight loss.

The present study had some limitations. First, there is a risk of selection bias due to the retrospective design. Second, nutritional status was evaluated only by body weight and serum albumin level. Third, QoL was not compared between the two groups, which is important in the palliative care setting. However, QoL is well known to fall immediately following SEMS insertion and is improved after PG feeding [7,10,29]. Our study also had strengths. To our knowledge, this is the first study to compare the outcomes between SEMS insertion and PG feeding in patients with esophageal cancer and dysphagia. Surprisingly, we observed a survival benefit in the gastrostomy group compared to the stent group. The gastrostomy group also had better nutritional status after the procedure than the stent group. In conclusion, our results suggest that PG feeding is a better option than SEMS insertion for patients with esophageal cancer and dysphagia.

## Supporting information

S1 File Dataset of this study. (XLSX)

## Abbreviations

CT: computed tomography
FCSEMS: fully covered self-expandable metal stent
HR: hazard ratio
PG: percutaneous gastrostomy
QoL: quality of life
RCT: randomized controlled trial
SEMS: self-expandable metal stent
